# Mandibular Ramus Fracture: An Overview of Rare Anatomical Subsite

**DOI:** 10.1155/2015/954314

**Published:** 2015-11-03

**Authors:** Anendd Jadhav, Bhushan Mundada, Rahul Deshmukh, Umesh Bhutekar, Atul Kala, Kapil Waghwani, Apoorva Mishra

**Affiliations:** Department of Oral and Maxillofacial Surgery, Sharad Pawar Dental College and Hospital, Wardha, Maharashtra 442004, India

## Abstract

*Aim*. The present study aims at exemplifying the incidence, and aetiology and analyses the outcomes of open reduction internal fixation (ORIF) over closed treatment of mandibular ramus fractures. *Patients and Method*. In the present retrospective analysis of mandibular fracture patients, variables analysed were age, sex, cause of injury, pretreatment occlusion, treatment given, period of maxillo-mandibular fixation (MMF), and posttreatment occlusion. *Results*. Out of 388 mandibular fractures treated, ramus fractures were 12 (3.09%). In the present study, predominant cause of mandibular ramus fracture was road traffic accident (RTA) *n* = 07 (58.33%) followed by fall *n* = 04 (33.33%) and assault *n* = 1 (8.33%). The average age was 35.9 years with a male predilection. Of these, 9 patients were treated with ORIF while remaining 3 with closed treatment. The average MMF after closed treatment was 21 days and 3 to 5 days after ORIF. There was improvement in occlusion in all 12 patients posttreatment with no major complication except for reduced mouth opening in cases treated with ORIF which recovered with physiotherapy and muscle relaxants. *Conclusion*. Mandibular ramus fractures accounted for 3.09% with RTA as a common aetiology. ORIF of ramus fractures facilitated adequate functional and anatomic reduction with early return of function.

## 1. Introduction

Since the earliest report on mandibular fractures dating back to 1650 BC in Egypt [1], oral and maxillofacial surgeons have studied the pattern of mandibular fractures without reaching a consensus on the most common pattern. This is because epidemiologic surveys have varied with geographic region, population density, socioeconomic status, regional government, educational status of the population studied, and era [2].

Despite the fact that the mandible is the largest and strongest facial bone, it is very frequently fractured (second to nasal bone fractures) as a result of the prominent and exposed position of the head. It is generally occurring 2 to 3 times as often as midfacial fractures [3]. In contrast to this opinion was the study by Scherer et al. [4] who found that mandibular fractures accounted for only 15.5% of all facial fractures.

The mechanism of injury correlates with the anatomical location of the mandibular fracture with defined anatomic fracture patterns. A motor vehicle crash or fall with an anterior impact results in symphyseal, parasymphyseal, and condylar fractures, whereas a lateral impact will result in angle, body, and contralateral condylar fracture [5]. Amongst the various anatomical subsites, the incidence of coronoid, alveolar, and ramal fracture is very low. Ramus fractures rank as the third least common fracture after coronoid and alveolar fracture [6] and this has been closely endorsed by others [7, 8].

The reported incidence of mandibular ramus fracture varies unpredictably within India and other Asian countries. In India, Barde et al. evaluated the pattern of mandibular fractures in central India and reported an incidence of 5.5% of mandibular ramus fractures attributing RTA as the major etiological factor [9]. On the contrary, Subhashraj et al., found it to be 3.0% in the southern state of Chennai [8] whereas Kumar et al. presented the lowest incidence of 0.9% in the Goan population depicting a wide variability of this rare entity in the Indian population itself [10]. Amongst the other Asian countries Almasri reported a lower incidence of 1.35% in the Saudi population [11] whereas it reported 3.19% in Kuwaiti population [12].

Anatomically, ramus is draped by masseter buccally, medial pterygoid muscle lingually, and pterygomasseteric sling at lower border, which facilitates minimum displacement of ramus after it gets fractured. Because of this obvious reason, most of surgeons manage this fracture by closed treatment. However, there are certain hostile limitations of closed reduction like prolonged maxillomandibular fixation (MMF), nonmaintenance of oral hygiene, risk of airway compromise, noncompliance of a patient, deprived of nutrition, and delayed recovery [13].

The present study aims at exemplifying the incidence and aetiology and analyses the outcomes of open reduction and internal fixation (ORIF) over closed treatment of these fractures.

## 2. Patients and Method

A retrospective analysis was conducted of the medical records of all trauma patients who presented with signs and symptoms associated with mandibular fractures on presentation to Department of Oral and Maxillofacial Surgery, Acharya Vinoba Bhave Rural Hospital, Datta Meghe Institute of Medical Sciences, from May 2010 through May 2015. The data were obtained by reviewing clinical case record sheets and imaging records. The present study was exempted from institutional ethical committee clearance on the grounds of retrospective observational nature of study and nondisclosure of patient's identity.

**Figure 1:**
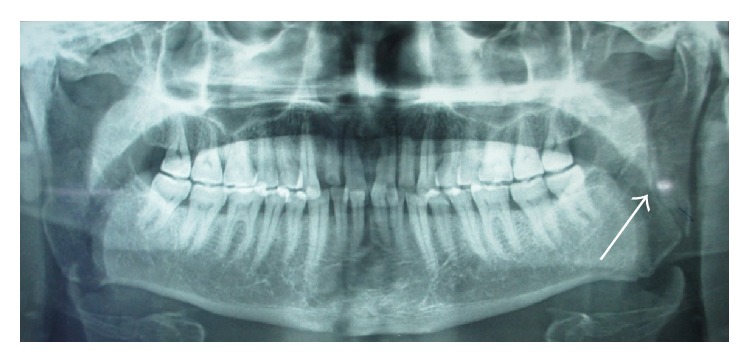
Fracture line running vertically from the sigmoid notch to the posterior border of the mandible, considered in ramus fracture.

**Figure 2:**
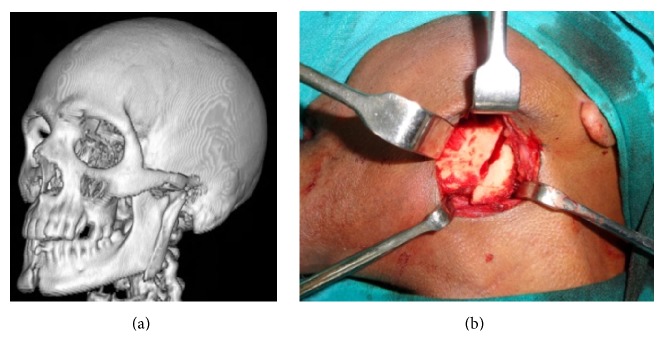
Fracture line extending from sigmoid notch vertically downwards to lower border of mandible, considered in ramus fracture.

Structurally, the area between the subcondyle and angle of mandible is considered as ramus of mandible. Essentially, fracture lines pass through these areas, for instance, line either running obliquely from sigmoid notch to the posterior border of mandible (Figure 1), running horizontally from anterior border to posterior border of mandible, or running from coronoid process to posterior border of mandible. Furthermore, fractures extending vertically downwards from sigmoid notch to the lower border of mandible (Figure 2) were included as a ramus fracture. The primary outcome variables were the treatment given, period of MMF, and posttreatment occlusion, while the secondary outcome variables were age, sex, and the cause of injury.

## 3. Observation and Results

The total number of patients with maxillofacial trauma reported in period during May 2010 to May 2015 to our department was 957, out of which mandibular fracture cases were 409 (42.73%) in number. Of these, only 388 patients underwent the treatment, while the other 21 patients either refused treatment or they were elected to be treated elsewhere and so were excluded from study.

Out of 388, patients with ramus fractures were 12 in number which accounted for 3.09% of mandibular fractures. The age ranges of these patients were from 22 to 48 years with the average age being 35.9 years. Reported cases show male predilection, with ratio of male : female as 5 : 1 (10 males and 2 females). The characteristics of the study population have been summarised in Table 1.

In the present study, causes for ramus fractures were varied; the predominant primary causative factor was RTA *n* = 07 cases (58.33%) followed by fall *n* = 04 (33.33%) and assault *n* = 1 (8.33%).

Pretreatment occlusion for all 12 patients was deranged, of these 9 patients were treated with open reduction and internal fixation while the remaining 3 patients were treated with closed reduction. In cases of closed treatment of ramal fractures, the concomitant fractures were treated by ORIF as per the standard operative protocol. The average MMF after closed reduction was 21 days and 3 to 5 days after open reduction. There was improvement in occlusion in all 12 patients after treatment and there was no major complication reported in any of the cases except for reduced mouth opening in cases treated with open reduction which was recovered with physiotherapy exercises and muscle relaxants.

## 4. Discussion

The sheer pace of modern life with high-speed travel as well as an increasingly violent and intolerant society has made facial trauma a form of social disease from which no one is immune [14]. The present study being a retrospective analysis from a single tertiary care trauma centre and with a limited sample size attempts to highlight mandibular ramus fractures and its management adding to the existing literature.

The incidence and causes of maxillofacial injuries show that patterns of maxillofacial fractures have changed over the decades and continue to do so [15]. Mandible being a U shaped bone, its fractures are often multiple. Most surveys show that just fewer than 50% are isolated, the same amounts are doubly fractured, and a small percentage has more than 2 fractures [16]. There exists a geographic variation in the pattern of mandible fractures. Main causes worldwide are traffic accidents, assaults, falls, sports-related injuries, and civil warfare.

Regardless of geographic location, most fractures occur in men (67 to 88%) aged 25 to 34 years [17]. As men age, they are less likely to sustain mandible fractures from interpersonal violence or a motor vehicle crash and more likely to do so as a result of a fall. Approximately 25% of mandible fractures in women are attributable to falls. These results should be interpreted with caution because the mechanisms of injury and anatomical locations of the fractures are not always consistent, suggesting a potential underreporting of domestic violence [18]. The results of our investigation are largely in agreement with those of previous reports [8, 19, 20] particularly with regard to age and sex.

Many authors have reported motor vehicle accidents as a major cause of facial injuries. Our study concurs with the findings of the previous investigators [17, 21], where RTAs were the single most frequent cause of mandible fracture. In the present study, RTA 58.33%, followed by fall 33.33%, constitute the factor responsible for ramus factors, whereas fall was common only in the elderly age group [22].

The incidence of ramus fractures was 3.09% of all mandibular fractures reported at our centre. This is in concurrence with findings of others [7, 8, 12] while Kumar et al. reported a much lower incidence of 0.9% [10]. The lower reported incidence of ramus fractures in the literature may be due to its potential to remain undiagnosed because of muscular enveloping by pterygomasseteric sling on either side which prevents its severe displacement and lack of univocal unanimity for the exact location of its structural position between condyle and angle of mandible.

The aim of mandibular fracture treatment is the restoration of anatomic form, function with particular care to reestablishment of occlusion, and facial aesthetics, which is often contingent on a precise bony reduction and immobilisation. A less precise bony reduction may be acceptable if there are no opposing teeth or in an edentulous mandible. This can be achieved with MMF alone or in combination with surgical exposure and internal fixation. The controversy of treating ramus fractures by either ORIF or closed treatment can be debated and discussed at length as there is lack of evidence based literature for its management till date.

Mandibular ramus is located between dentate (angle/body) and nondentate (condyle and coronoid) part of mandible. There are no clear indications and contraindications about open or closed treatment of these fractures. Management of these fractures is still an enigma; however, certain aspects of treatment remain amenable to personal opinions and clinical impression. As this fracture seldom causes occlusion derangement and due to difficulty in access to fracture they are conventionally managed by closed treatment [23]. Surgeons hesitantly opt for ORIF treatments mainly because of troublesome surgical exposure, particularly by the proximity of facial nerve branches. Inevitable scars caused by cutaneous incision, risks of facial palsy, and difficulty of incorporating technological innovations, with long-term learning curves and extended operating time, account for some of the drawbacks related to operative interventions.

An alternative to extra oral approach is the use of “transbuccal” approach, wherein exposure of fracture site and reduction of fracture is done predominantly via intraoral approach and a percutaneous stab incision is given extraorally in the cheek to facilitate the insertion of transbuccal trocar achieving lateral plating for which screws are fixed through the transbuccal cannula. The primary advantage of this technique is minimal scar; however, despite this aesthetic advantage, it has some inherent disadvantages like requirement of specialized armamentarium and the long learning curve as it is a technique sensitive procedure. Furthermore, its clinical applicability in ramus fracture still remains to be investigated.

**Figure 3:**
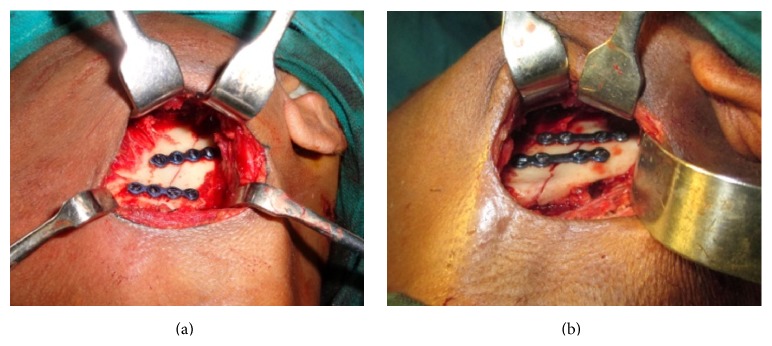
Surgical access by Risdon's approach and fixation with two noncompression mini plates.

**Figure 4:**
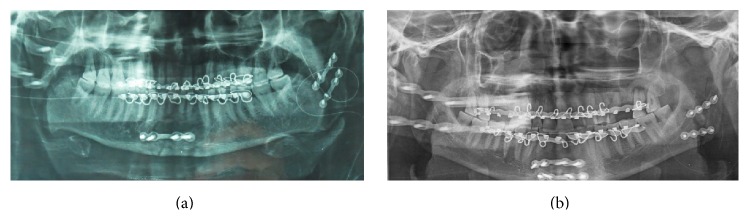
Postoperative check radiographs showing fixation of mandibular ramus fracture by two noncompression mini plates.

Ramus fractures are seldom lonely. In majority of the documented cases (except for one), ramus fractures were associated with other mandibular fractures and/or with midface fractures. There was no predictable pattern of associated fractures observed in our study. Of the 12 cases, 09 were treated by ORIF wherein the site was accessed by Risdon's incision (Figure 3) and fixated by two noncompression mini plates (Figure 4). These are preferred owing to its larger cross-sectional area to avoid torqueing and splaying at either ends of fracture which might occur under the influence of the muscles attached.

Open reduction and rigid internal fixation provide a number of advantages like functional as well as anatomical reduction and immobilization of the fracture, early return to function, easier maintenance of oral hygiene, improved nutrition, and reduced risk of airway compromise. All of the cases treated by ORIF and closed treatment had satisfactory, stable occlusion at the conclusion of the treatment without any major complications.

The present report on clinical potential of mandibular ramus fracture and analysis of its management is limited because the study is from a single institution which compromises results in retrospective manner. Providing clear treatment recommendations on the basis of a series of 12 patients is challenging. The observation of this study should be supported by further well designed elaborative randomized controlled studies, comparing open versus closed treatment and also different modalities of open treatment with each other, for substantial evidence based management of these fractures.

## 5. Conclusions

Within the limitations of the current study, we conclude that ramus fracture is a relatively rare subsite to get fractured amongst mandible fractures. ORIF of ramal fractures by two noncompression mini plates confers adequate anatomical, functional reduction comprising length, alignment, and rotational axis of adjacent fracture fragments, and immobilization with good outcomes and relatively early return to function in our small series.

## Figures and Tables

**Table 1:** Characteristics of study population.

Sr. number	Age (Years)	Sex	Cause of injury	Concomitant fractures	Treatment given
1	23	M	Assault	Symphysis	ORIF
2	46	F	Fall	Zygomatic arch	Closed treatment
3	29	M	RTA	Sub condylar	Closed treatment
4	33	M	RTA	ZMC, symphysis	ORIF
5	48	M	Fall	Parasymphysis and contralateral angle	ORIF
6	26	M	RTA	Symphysis	ORIF
7	24	M	RTA	Le Fort II	ORIF
8	30	M	RTA	Contralateral parasymphysis	ORIF
9	52	M	Fall	Parasymphysis	ORIF
10	27	M	RTA	None	Closed treatment
11	44	M	RTA	Le Fort I, contralateral parasymphysis	ORIF
12	49	F	Fall	ZMC, parasymphysis	ORIF
